# Rare diseases and disabilities: improving the information available with three Orphanet projects

**DOI:** 10.1186/1750-1172-9-S1-O31

**Published:** 2014-11-11

**Authors:** Myriam de Chalendar, Marie Daniel, Annie Olry, Ana Rath

**Affiliations:** 1 Orphanet, Inserm US14, Paris, France

## 

There is currently very little information available about the disabilities encountered by rare disease (RD) patients. Orphanet [http://www.orpha.net], the international database and portal on RDs and orphan drugs, has developed various projects to improve the knowledge and visibility of disabilities associated with RDs, and to provide tools to help the stakeholders.

First, we have added content to the texts of the Orphanet Encyclopaedia for the General Public (133 texts) about the daily difficulties associated with the disease and its management. This information is provided through three questions: “What disabilities result from the disease?”, “What resources are available to limit and prevent the disability?” and “Living with: the disability on a daily basis”. These texts are validated by medical experts, disability specialists and patient support groups.

Secondly, we have created a specialised collection of texts dedicated to professionals and social service providers, the Orphanet Disability Encyclopaedia. It focuses on the disabilities associated with a specific RD. These disability factsheets provide a brief overview of the medical aspects of the disease, validated by medical experts, and include a description of the disabilities experienced by patients and their management. Fifteen texts are currently available in French.

Finally, with the Orphanet Disability Project, we index the functional consequences of each RD with the Orphanet Functioning Thesaurus, adapted from the “Activities and participation” and “Environmental factors” domains of the International Classification of Functioning, Disability and Health-Children & Youth version (ICF-CY [1]), as well as additional terms to describe cognitive abilities, sleep, temperament and behaviour. Through a questionnaire sent to medical experts, disability specialists and patient organisations, we collect data for each RD: the activity limitations and participation restrictions, their temporality during the course of the disease (permanent or transient difficulty, delay, loss of abilities), their severity and respective frequency in the patient population with current standard management, and important environmental factors. The collected data is analysed and standardised to constitute the Orphanet Functioning Database. 857 RDs are already indexed and 540 more are in progress, thanks to the contribution of hundreds of people and organisations from 43 countries. These RD disability core sets, which can be integrated into information systems, will be freely available in 7 languages. In addition, we will map the “Body structures” and the “Body functions” domains of the ICF-CY to the Human Phenotype Ontology [2], enabling us to list the anatomical structures and physiological functions impaired for each RD.

This information will increase knowledge and aid in better evaluating and managing the daily difficulties and needs experienced by RD patients. It can also help social agencies in distributing appropriate disability compensation measures with equity and equality. Finally, it will enable decision makers to assess the social burden of RDs and can be utilised in the set-up of measures that will allow for the better social integration of disabled people with RDs.
